# Intensified treatment with high dose Rifampicin and Levofloxacin compared to standard treatment for adult patients with Tuberculous Meningitis (TBM-IT): protocol for a randomized controlled trial

**DOI:** 10.1186/1745-6215-12-25

**Published:** 2011-02-02

**Authors:** Dorothee Heemskerk, Jeremy Day, Tran Thi Hong Chau, Nguyen Huy Dung, Nguyen Thi Bich Yen, Nguyen Duc Bang, Laura Merson, Piero Olliaro, Thomas Pouplin, Maxine Caws, Marcel Wolbers, Jeremy Farrar

**Affiliations:** 1 Hospital for Tropical Diseases Oxford University Clinical Research Unit, Wellcome Trust Major Overseas Programme 190 Ben Ham Tu, District 5, Ho Chi Minh City, Vietnam; 2 Hospital for Tropical Diseases, 190 Ben Ham Tu, District 5, Ho Chi Minh City, Vietnam; 3 Pham Ngoc Thach Hospital and, 120 Hung Vuong, District 5, Ho Chi Minh City, Vietnam; 4 Centre for Tropical Medicine, Nuffield Department of Medicine, University of Oxford, Oxford, UK

## Abstract

**Background:**

Tuberculous meningitis is the most severe form of tuberculosis. Mortality for untreated tuberculous meningitis is 100%. Despite the introduction of antibiotic treatment for tuberculosis the mortality rate for tuberculous meningitis remains high; approximately 25% for HIV-negative and 67% for HIV positive patients with most deaths occurring within one month of starting therapy. The high mortality rate in tuberculous meningitis reflects the severity of the condition but also the poor antibacterial activity of current treatment regimes and relatively poor penetration of these drugs into the central nervous system. Improving the antitubercular activity in the central nervous system of current therapy may help improve outcomes. Increasing the dose of rifampicin, a key drug with known poor cerebrospinal fluid penetration may lead to higher drug levels at the site of infection and may improve survival. Of the second generation fluoroquinolones, levofloxacin may have the optimal pharmacological features including cerebrospinal fluid penetration, with a ratio of Area Under the Curve (AUC) in cerebrospinal fluid to AUC in plasma of >75% and strong bactericidal activity against *Mycobacterium tuberculosis*. We propose a randomized controlled trial to assess the efficacy of an intensified anti-tubercular treatment regimen in tuberculous meningitis patients, comparing current standard tuberculous meningitis treatment regimens with standard treatment intensified with high-dose rifampicin and additional levofloxacin.

**Methods/Design:**

A randomized, double blind, placebo-controlled trial with two parallel arms, comparing standard Vietnamese national guideline treatment for tuberculous meningitis with standard treatment *plus *an increased dose of rifampicin (to 15 mg/kg/day total) and additional levofloxacin. The study will include 750 patients (375 per treatment group) including a minimum of 350 HIV-positive patients. The calculation assumes an overall mortality of 40% vs. 30% in the two arms, respectively (corresponding to a target hazard ratio of 0.7), a power of 80% and a two-sided significance level of 5%. Randomization ratio is 1:1. The primary endpoint is overall survival, i.e. time from randomization to death during a follow-up period of 9 months. Secondary endpoints are: neurological disability at 9 months, time to new neurological event or death, time to new or recurrent AIDS-defining illness or death (in HIV-positive patients only), severe adverse events, and rate of treatment interruption for adverse events.

**Discussion:**

Currently very few options are available for the treatment of TBM and the mortality rate remains unacceptably high with severe disabilities seen in many of the survivors. This trial is based on the hypothesis that current anti-mycobacterial treatment schedules for TBM are not potent enough and that outcomes will be improved by increasing the CSF penetrating power of this regimen by optimising dosage and using additional drugs with better CSF penetration.

**Trial registration:**

International Standard Randomised Controlled Trial Number ISRCTN61649292

## Background

Driven in part by the HIV epidemic, tuberculosis (TB) is a major global health problem. Of all the syndromes caused by *Mycobacterium tuberculosis *(Mtb), tuberculous meningitis (TBM) is the most severe. Almost all patients with untreated TBM die. Since the introduction of antibiotic treatment with streptomycin for TB in the 1950's the death rate has declined [1]. However morbidity and mortality overall are still high. In Vietnam the death rate in HIV negative patients treated according to current guidelines is 25%, and a further 30% of patients suffer long term neurological sequelae [2]. 50% of adult patients who are admitted with TB meningitis in Ho Chi Minh City are HIV positive. HIV significantly worsens outcome, with a mortality rate of 67% [3]. In our previous studies, the majority (75%) of deaths occurred within 1 month of starting treatment, and almost all deaths occurred within 6 months [3]. Across the globe similar high rates of mortality and disability are reported for TBM [4]. Although TBM predominantly occurs in developing countries, 320 TBM cases were reported in the UK in 2007 making it one of the three leading causes of meningitis in the UK. These numbers are likely to rise, due to the global increase of drug resistant TB, increasing use of immunosuppressive therapies, and the HIV epidemic. Along with increased awareness and vigilance for the disease, it is of the utmost importance to improve treatment for TBM based on sound scientific evidence.

### Pathogenesis of TBM

The exact pathogenesis is still largely unknown and current knowledge is based on original pathological studies performed in the early 1930's by Rich and McCordock who postulated that the development of TBM is a two step process [1]. The first step is believed to be a short bacteraemia following pulmonary infection that enables the mycobacteria to seed elsewhere in the body, including the meninges and brain parenchyma. Small foci are believed to form subpially or subependymally, the so called Rich foci. The second step is the rupture of these foci in the subarachnoid space, causing the onset of meningitis. Three general processes are thought to cause the subsequent neurological pathology; adhesion formation, obliterative vasculitis, and encephalitis or myelitis. Immunopathology of TBM is still poorly understood. As a result of a large randomized controlled trial in Vietnam [2], guidelines now recommend all patients with TBM should receive corticosteroids. This trial showed a 31% reduction in risk of death. TB treatment is complicated by a significant risk of adverse events, in particular liver toxicity [5]. In the patient group receiving dexamethasone there was a significantly lower incidence of adverse events compared with placebo. Even though adjunctive immunomodulatory treatment has a beneficial impact on outcome, mortality is still high, especially for the intermediate and high grade severity groups (MRC grade 2 and 3) and HIV co-infected patients. The host-response must be supplemented with appropriate anti-mycobacterial agents in order to facilitate rapid bacterial clearing and prevent a cascade of intracerebral events that will lead to clinical deterioration.

### Treatment of TBM

There is a lack of good quality evidence on TBM treatment, in particular for the anti-tubercular chemotherapy regimens. In the past decades, no randomized controlled trials have been published which compared different anti-tubercular regimens and there are no good quality cohort studies. Treatment schedules for TBM globally are not uniform and are mostly extrapolated from those used for pulmonary TB. The National Institute for Health and Clinical Excellence UK (NICE) has published guidelines for the UK in collaboration with the British Thoracic Society (BTS) in 2006, acknowledging the lack of evidence NICE classify the recommendation as Level 4 which indicates a weak evidence-base.

The current treatment guidelines for TBM in Vietnam recommend treatment in the intensive phase with rifampicin (10 mg/kg max 750 mg/day), isoniazid (5 mg/kg, max 300 mg/day), pyrazinamide (25 mg/kg, max 2 g/day) and streptomycin (20 mg/kg, max 1 g/day) for 3 months. All drugs are given orally once daily, with the exception of streptomycin which is administered intra-muscularly. This is followed by rifampicin and isoniazid for 6 months in the consolidation phase. In HIV-positive patients, streptomycin is replaced with ethambutol (15-20 mg/kg, max 1.2 g/day). However the variability of CSF penetration of the different first-line TB-drugs may warrant a need for adjustment of these regimens accordingly. In particular, the penetration of rifampicin, the key drug, is poor, as is that of ethambutol [6-10]. The mortality rate of TBM patients may reflect both poor antibacterial activity of current treatment regimes and poor penetration of these drugs into the central nervous system. For HIV patients the excess death rate may be a reflection of even less efficient uptake of antimycobacterial drugs due to malabsorption and subsequent low drug levels [9-11], combined with a severely impaired immune system. Improving the sterilising power of current therapy may result in improved outcomes of all TBM patients.

We propose a randomised placebo controlled trial to test this hypothesis in patients with TB meningitis. The study will compare standard anti-tuberculous treatment with anti-tuberculous treatment intensified with high dose rifampicin and levofloxacin.

### High Dose Rifampicin for TBM

Rifampicin is a semisynthetic derivative of rifamycin and is a key drug in the treatment of all forms of TB, demonstrated by the fact that in tuberculous meningitis resistance to this drug is associated with high rates of relapse and death [12]. Rifampicin is used throughout the whole of the 9 month treatment period in TBM. The recommended dose is 10 mg/kg/day. Dosage is administered according to weight categories. It is not recommended to divide tablets and hence many patients in these weight categories in fact receive less than the recommended 10 mg/kg.

Rifampicin penetrates well into cells, and is active against intra-cellular bacteria, but CSF concentrations are reported to be low [13-15]. There are few data comparing the Area Under the Curve (AUC) in the cerebrospinal fluid and plasma compartments [AUC_c_/AUC_p_], but the ratio is probably in the order of 10-20%. Penetration may be influenced by the level of damage to the blood brain barrier and the serum protein binding of rifampicin which approaches 80% [14].The therapeutic range of rifampicin lies between 8 - 24 μg/ml and levels below 4 μr/ml are considered very low [7,9]. Huge inter-individual variation in metabolism and rifampicin drug-levels have been reported, with worrying numbers of patients with low to very low levels. HIV infection has been associated with even lower plasma levels of Rifampicin [10,11]. Consequently CSF levels are expected to be in the low or very low ranges.

Recently an Indonesian study has been published concluding that a dose increase from 10 to 13 mg/kg/day is associated with a 65% increase in mean plasma AUC_0-24 h _and 49% increase in plasma C_max _without a significant increase in the rate of serious adverse events [16,17]. This study was not powered for outcome, but the increase in the drug levels in the CSF associated with a relatively small increase in the doses are fascinating and may be of clinical importance for TBM patients.

Rifampicin is relatively non-toxic. The most noticeable side effect is red staining of body secretions, also known as the "red man syndrome" [15]. Other side effects include rash, flushing and gastrointestinal disturbances (usually mild). Drug-induced hepatitis (DIH) is a well recognised side-effect of TB treatment, with a frequency of between 5 and 33% [5]. The drugs most usually implicated are isoniazid and pyrazinamide. However, transient elevation of transaminases (and less commonly bilirubin) is reported with rifampicin use. DIH usually responds well to treatment interruption. A gradual sequential re-introduction of each drug is usually tolerated without recurrence of hepatitis [5].

Based on the data presented in this section we propose an increased dose of rifampicin of 15 mg/kg for patients with TBM, to increase serum levels and we anticipate increase levels of rifampicin at the site of infection. With this strategy we aim to improve the sterilising power of the anti tubercular regimen in the brain.

### Levofloxacin for TBM

Of the anti-tubercular reserve drugs, fluoroquinolones, in particular levofloxacin, are an attractive candidate in the treatment of TBM. Especially the later generation drugs such as levofloxacin, moxifloxacin and gatifloxacin have improved *in vitro *activity, and there is evidence of good sterilising activity in sputum in pulmonary TB [18,19]. Despite demonstration of *in vitro *activity of various drugs against Mtb, there has been little progress in drug development or assessment of alternative anti-mycobacterial treatment regimes in TBM [20]. Trials in pulmonary TB however, have demonstrated the safety of prolonged treatment with fluoroquinolones [21,22].

Fluoroquinolones are an attractive option for the treatment of TBM because of their demonstrable *in vitro *activity, tolerability, good bioavailability and ease of administration [23-37]. Our centre recently completed a pharmacokinetic study comparing oral ciprofloxacin (750 mg/12 hours), levofloxacin (500 mg/12 hours) or gatifloxacin (400 mg/24 hours) for the first 60 days in patients with TBM, and examining their pharmacokinetic interaction with rifampicin. We found levofloxacin to have excellent CSF penetration, with AUC_c_/AUC_p _= 75%. This compared favourably with gatifloxacin (35%) and ciprofloxacin (14%). Of the second generation fluoroquinolones, levofloxacin has the greatest Early Bactericidal Activity (EBA), comparable to that of isoniazid. The MIC of drug sensitive isolates is in the order of 0.25 - 1 μg/ml [34,38]. Plasma levels of levofloxacin in Vietnamese patients are comfortably in excess of this, with AUC_0-12 _of 80 mg/hr/L (G. Thwaites Personal Communication).

Fluoroquinolone resistance has been identified in strains from Vietnam, but currently is rare in TBM cases (<1%) and less frequent than rifampicin resistance (M. Caws Personal Communication). Levofloxacin has performed well in human studies using surrogate markers of efficacy such as EBA (rate of fall of colony forming units in sputum) [18]. This is probably a reflection of its favourable pharmacokinetic profile resulting in high plasma and intracellular concentrations.

Levofloxacin has the advantages of a favourable toxicity profile, affordable cost and an extensive amount of available safety data from clinical trials examining its prolonged use in pulmonary TB. We propose levofloxacin as an additional drug in the highly active treatment arm combined with a high dose of rifampicin in this randomised placebo controlled trial.

### Hypothesis

Current antimycobacterial regimes are not potent enough to treat TBM effectively, as most of the antimycobacterial drugs have very low cerebrospinal fluid penetration. Increasing levels of effective anti-mycobacterial drugs in the cerebrospinal fluid and hence at the site of infection will we hope improve treatment outcome.

### Aims

The primary aim of this study will be to reduce mortality by intensifying the induction phase of anti-tuberculous treatment of TBM. Secondary aims are to assess the effect on morbidity and disability of intensifying standard treatment, to assess the safety and tolerability of the intensified treatment.

## Methods/Design

### Design

**Figure 1:**
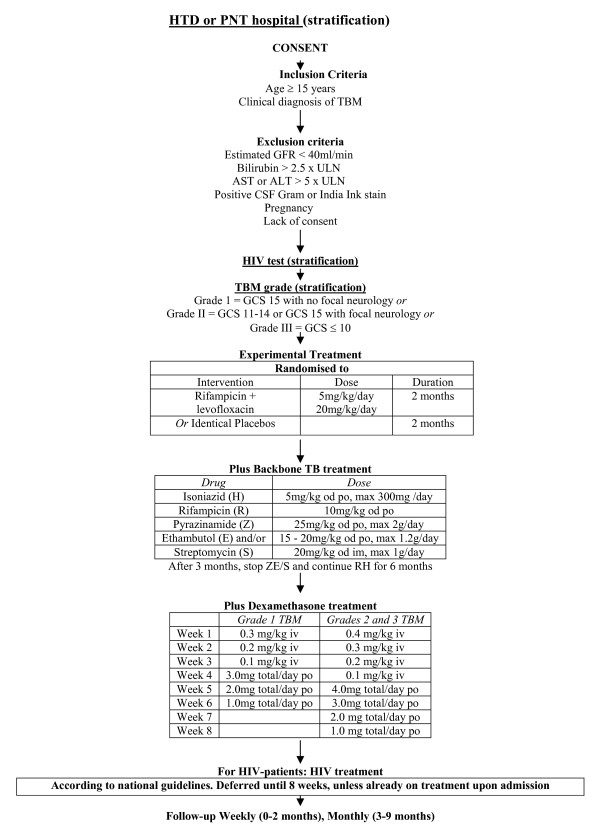
**Trial flow diagram**.

This is a randomized, double blind, placebo-controlled trial with two parallel arms, comparing standard anti-tubercular treatment for tuberculous meningitis (according to national guidelines) with standard treatment *plus *an increased dose of rifampicin and additional levofloxacin. We aim to enhance the antimycobacterial efficacy of current treatment for TB meningitis in Vietnam by adding levofloxacin 20 mg/kg/day to the intensive phase of treatment and increasing the dose of rifampicin to 15 mg/kg/day during the intensive phase of treatment for the duration of 2 months (Figure 1)

### Inclusion and exclusion criteria

All adult patients (aged ≥ 18 years) with a clinical diagnosis of TBM (Additional File 1) presenting to the Hospital for Tropical Diseases (HTD), HCMC, or Pham Ngoc Thach Hospital (PNT), HCMC, will be eligible to enter the study. Exclusion criteria are: a positive CSF Gram or India Ink stain, pregnancy, known hypersensitivity/intolerance to fluoroquinolones or rifampicin, creatinine >3 ULN, laboratory contraindications to antituberculous therapy (bilirubin > 2.5 × ULN, AST or ALT > 5 × ULN), diagnosis of multi-drug resistant TBM or lack of informed consent

### Primary endpoint

The primary endpoint will be overall survival, i.e. time from randomization to death during a follow-up period of 9 months. Survivors will be censored at the date they were last known to be alive (i.e. date of last follow-up visit, loss to follow-up or withdrawal).

### Secondary endpoints

The secondary endpoints are:

a) neurological disability at 9 months, assessed using the "two simple questions" and Rankin score (Additional File 2).

b) time to new neurological events or death (Neurological events are defined as: any of the following adverse events: cerebellar symptoms, coma, hemiplegia, neurological deterioration, paraplegia, seizures, cerebral herniation or cranial nerve palsy or a fall in Glasgow coma score by ≥ 2 points for ≥ 2 days from highest previously recorded Glasgow coma score).

c) any grade 3 or 4 adverse event (Additional File 3).

d) rate of treatment interruption for adverse events.

e) the rates of asymptomatic transaminitis and symptomatic hepatitis.

f) time to new or recurrent AIDS defining illness or death (in HIV positive patients only).

g) time to undetectable viral load (in HIV positive patients only).

h) CD4 count at completion of therapy (in HIV positive patients only).

### Randomization procedure

Randomization will be 1:1 and patients will be stratified according to hospital site (HTD and PNT), HIV status and TBM disease severity at presentation (TBM severity will be graded according to the modified MRC system, Additional File 4). Enrolment logs specific to site, HIV positivity and severity of TBM will be used to assign patients to the next available sequential number within the appropriate stratification group. The assigned number will correspond to two pre-packaged bottles which contain a 2 month supply of additional doses of rifampicin and levofloxacin or visually matched placebos of each. Bottles will be prepared centrally by an unblinded study pharmacist and distributed to the sites in batches as required. Only two central study pharmacists who will hold the master randomization list will know the contents of each bottle. This list will be accessed only in the case of emergency unblinding authorized by an investigator as per standard operating procedures. Within strata, we will use block randomization with variable block size. Stratified randomization will ensure that almost equal numbers of patients with equivalent prognosis are included in the two treatment arms.

### Enrolment and blinding

For each group, tablets of intensified treatment or placebo will be placed in bottles in coded sealed packages, which are labeled with the randomization number of the patient. Drug appearance and administration schedules will be identical to maintain blinding amongst the attending physicians and nurses. The admitting physician will be responsible for ensuring the patient satisfies the entry criteria, completes informed consent and starts a study drug treatment package. Clinical details will be recorded in individual patient case record forms (CRFs).

### Additional treatment for all TBM patients

All patients will receive backbone treatment with standard antituberculous therapy (Additional File 5) and adjunctive dexamethasone (Additional File 6) on study entry, according to Pham Ngoc Thach Hospital and Vietnamese National TB Programme guidelines. All patients receiving isoniazid will also receive pyridoxine (vitamin B6). Patients, who develop TBM while on treatment for pulmonary TB, will be eligible to enter the study. According to Vietnamese hospital guidelines, these patients will receive TBM-treatment with 5 first line TB-drugs (SRHZE) which will be the "backbone" or standard TB treatment. If patients consent to take part in the trial, they will be randomized to intensified TB treatment or placebo as described previously. If the patient is comatose, the drugs can be given by nasogastric tube.

### Second-line antituberculous therapy

Patients with a definite or clinical diagnosis of multi-drug resistant (MDR) TBM will be excluded from the trial and referred to the MDR-TBM department for second- line MDR treatment according to NTP guidelines. Change to: Patients who have been randomized and are subsequently diagnosed with MDR-TB will be referred for second-line therapy according to Vietnamese guidelines. They will continue to be followed up in the study and included in the ITT analysis.

### Anti-retroviral therapy

Antiretroviral therapy will be provided for HIV infected patients within the current Vietnamese guidelines. Antiretroviral therapy is available free of charge through the US Government PEPFAR programme for in-patients with life-threatening opportunistic infections from 2 weeks after admission. HIV positive patients will be referred to the HIV Outpatient Clinic (OPC). To ensure that treatment naïve HIV-positive patients receive ARV treatment at 8 weeks and continue their treatment, patients will be enrolled either at the hospital OPC or through local specialized OPC services, following standard local practice. For ARV-treatment naïve patients, ARV therapy will be initiated after 8 weeks of TB therapy. This is consistent with the results of the recent trial of immediate or deferred antiretroviral therapy in TB meningitis, carried out by our group and consistent with local practice guidelines (E. Torok Personal Communication). There are currently 4 different treatment schedules for first line ARV treatment in Vietnam, all containing 2 NRTI's and 1 NNRTI. Patients already receiving ARVs at the time of diagnosis of TBM will continue ARV therapy. The majority of patients will be on schedules containing nevirapine (NVP). According to Vietnamese guidelines NVP will be changed to efavirenz for HIV positive patients that require a TB-regimen containing rifampicin.

Reports show good clinical outcome for patients on a 600 mg dose of efavirenz who are on TB-regimens containing rifampicin [39]. Accordingly and following National treatment guidelines, the dose of efavirenz will not be increased for patients on TB-regimens containing rifampicin. Second line ARV treatment is rarely prescribed in Vietnam. Very few patients will have a PI in their treatment schedule. Decisions on dose or schedule adjustments for these patients will be made on an individual basis, following the National guidelines. Liver function tests will be monitored in all patients.

### Prophylaxis for opportunistic infections (for HIV positive patients)

Patients will receive prophylaxis for opportunistic infections according to Vietnamese national guidelines. If the CD4 count is less than 200 cells/uL, patients will receive prophylaxis against *Pneumocystis jirovecii *pneumonia and cerebral toxoplasmosis with cotrimoxazole 960 mg/day.

### Data on concomitant medications

At each visit, information on other medications, including start dates and indications, will be documented in the case record forms.

## Data collection

### Baseline evaluation

On admission all patients will have a full clinical assessment and examination to determine TBM MRC grade (Additional File 4), and assess any neurological symptoms and signs. The following laboratory tests will be performed at study entry: haematology (full blood count), biochemistry (total protein, albumin, creatinine and liver function tests), cerebrospinal fluid (cell count, protein, glucose, lactate, Gram stain, Ziehl - Neelsen (ZN) stain, India Ink stain, cryptococcal antigen, bacterial and mycobacterial culture), HIV test. Additional tests for HIV positive patients will include immunology (CD4 count) and virology (confirmatory HIV test, plasma HIV-1 RNA, HbsAg, HBV DNA, HCV Ab test, HCV RNA). A baseline chest radiograph will be performed for all patients. A CT or MRI brain scan will be performed if there is evidence of raised intracranial pressure or focal neurological abnormalities.

### In-patient monitoring

Patients will have daily review until discharge from hospital at 2 months (this period may be adjusted according to clinical findings) for neurological, drug-related adverse events (Additional File 3) and new or recurrent AIDS defining illnesses (HIV positive patients only). In-patients will have weekly routine laboratory monitoring of haematology (full blood count) and biochemistry (creatinine and liver function tests). Cerebrospinal fluid analysis will be done routinely according to local guidelines at 4 and 8 weeks.

A subgroup of patients recruited to the pharmacokinetics study will have additional blood and CSF samples taken. Other investigations may be performed if clinically indicated. Uniform management of patients and recording of data will be ensured by the principal investigator who will make a daily round of all study participants. Following discharge, patients will be followed up as part of the National Tuberculosis Programme. Formal outpatient review will occur monthly until the completion of treatment, at 9 months.

### Out-patient monitoring

Out-patients will pay monthly visits to the out-patient department (OPD) for clinical evaluation and laboratory monitoring until completion of treatment at 9 months. Haematology (full blood count) and biochemistry (creatinine, liver function tests) will be checked monthly. Final cerebrospinal fluid analysis will be at 9 months. HIV positive patients will have additional samples taken for immunology (CD4, CD8) and virology (plasma HIV-1 RNA) every 3 months until the end of treatment. A subgroup of HIV positive patients who started ARV-treatment at week 8 of TBM-treatment will have an additional cerebrospinal fluid analysis at the 3 month OPD-visit.

### Imaging

Chest and brain imaging will be performed as clinically indicated - i.e. in the event of pulmonary or neurological deterioration.

### Clinical trial specimens

All clinical trial specimens will be labeled with the patient's trial number. Samples will be transferred to the laboratories at the Hospital for Tropical Diseases and Pham Ngoc Thach Hospital for initial processing. Investigation results will be issued to the investigators in a timely manner and a hard copy of the results will be retained in the laboratory for verification. Samples will be stored securely in freezers at the Hospital for Tropical Diseases and Pham Ngoc Thach Hospital prior to transfer to the Oxford University Clinical Research Unit for further investigations and long term storage.

## Management of adverse events and toxicities

### Management of antituberculous toxicity

A symptom checklist will be used to determine clinical toxicity. Routine laboratory tests will be performed weekly as an inpatient and monthly as an outpatient. Clinicians may also request additional tests if clinically indicated. Common side effects of first-line TB-drugs are given in Additional File 3). Therapy may need to be interrupted for severe (grade 3 or 4) adverse events. Once clinical and laboratory features resolve, drugs may be reintroduced sequentially. Details of management are given in Additional Files 7,8,9

### Reporting adverse events

According to the ICH Guidelines for Clinical Safety Data Management: definitions and Standards for Expedited Reporting (1994), a serious adverse event (SAE) is defined as "any untoward medical occurrence that a) results in death, b) is life threatening, c) requires unplanned inpatient hospitalization or prolongation of existing hospitalization, d) results in persistent or significant disability/incapacity or is a congenital anomaly/birth defect, e) any other important medical condition, which, although not included in the above, may jeopardize the subject and may require medical or surgical intervention to prevent one of the outcomes listed."

If the patient dies or experiences an adverse event (serious, grade 3 or 4, or one leading to modification of treatment, see Additional File 3 Common Toxicity Criteria) the investigator should inform the principal investigator as soon as possible and complete the specific case report form. When applicable, adverse events will be treated as per the management guidelines in Appendix 2.0. All SAEs will be recorded on the SAE form and reported to the principal investigator, the Oxford Tropical Research Ethics Board and the Ethical Committee of the Ministry of Health Vietnam within 72 hours of the event. Unblinded adverse event and mortality summaries will be reviewed by the trial's independent Data and Safety Monitoring Committee at regular time points (see section "ethical issues" for details.) If there is a protocol violation for any reason this will be fully recorded. Protocol violations which affect patient safety will be reported to the Oxford Tropical Research Ethics Board and the Ethical Committee of the Ministry of Health Vietnam.

## Statistical considerations

### Sample size and power calculations

The trial is powered for the primary endpoint, i.e. overall survival during the 9 month follow-up period. Based on previous publications from our research group, the 9-month mortality in the control arm is expected to be 60-65% in HIV-positive and around 25% in HIV-negative TBM patients [2]. Approximately 50% of TBM patients in the participating hospitals are HIV-positive; we therefore expect an overall 9-month mortality rate of around 40% in the control arm of our trial. An absolute risk reduction of 10% in 9-month mortality from 40% to 30% due to intensified treatment was judged as both realistic and clinically relevant.

Assuming proportional hazards, these mortality estimates translate into a hazard ratio of 0.7 [= log(1-0.3)/log(1-0.4)], i.e. a 30% risk reduction due to intensified treatment on the hazard ratio scale. Based on Schoenfeld's formula, a total of 247 deaths are required to detect a hazard ratio of 0.7 based on a two-sided test at the 5% significance level with 80% power; assuming an overall mortality rate of 35% in the trial, this translates into 706 required patients. In order to account for potential deviations from our assumptions and losses to follow-up, a safety margin of 6% was added to this number leading to a total sample size of 750 patients (375 per treatment group).

HIV-positive TBM patients with a very high mortality are a particularly important subgroup of our study population and we aimed to have sufficient power to also detect a benefit in this subgroup of patients alone. If intensified treatment reduces 9-month mortality by 15% in HIV-positive patients (from 65% to 50%), corresponding to a hazard ration of 0.67, a total of 196 deaths in HIV-positive patients are required to detect this difference with 80% power; approximately 350 HIV-positive patients are necessary to observe 196 deaths during follow-up.

To guarantee both sufficient power in the subgroup of HIV-positive TBM patients and a sufficiently high event rate in the total population, the trial will continue recruitment until both a total of 750 patients and a minimum of 350 HIV-positive patients have been recruited.

### Primary and secondary endpoint analysis

The primary endpoint of this trial is overall survival, i.e. time from randomization to death, during the entire follow-up period of 9 months. Overall survival will be analyzed with a log-rank test stratified by HIV status (positive/negative) and TBM disease severity at presentation (modified MRC grade I, II or III). Kaplan-Meier plots and explicit survival estimates at 3, 6 and 9 months of follow-up will also be calculated for the full populations and in the subgroups defined by HIV status and TBM disease severity separately.

In a second stage, overall survival will be modeled using the Cox proportional hazards regression model and the following covariates (in addition to the treatment group): TBM disease severity (grade I, II, or III), HIV status (positive/negative), participating hospital (PNT/HTD), previous TB treatment (yes/no), drug resistance (drug sensitive/MDR-TB/Isoniazid resistant non-MDR). A separate analysis for HIV positive patients only will be performed which will include prior antiretroviral therapy (yes/no), CD4 cell count and log10-HIV viral load at baseline as additional covariates.

The homogeneity of the treatment effect on overall survival in the subgroups defined by TBM grade (I, II, or III), HIV status (positive/negative), prior TBM treatment (yes/no), drug resistance (drug sensitive/MDR-TB/isoniazid resistant non-MDR) respectively, will be examined and tested using tests of interaction between treatment and the grouping variable.

For the secondary endpoints concerning neurological disability, the disability score at month 3, 6, and 9 of follow-up is defined as the higher (worse) of the "simple question" and the Rankin score assessed at that time point as previously described [2]. Disability score will be defined as 4 (worst outcome) if the patient died prior to the respective time point. The score of primary interest is the month 9 score which will be compared between the two arms with the generalized Cochran-Mantel-Haenzsel test as described in Mantel's generalized statistics [40] taking into account that the disability score is ordinal. The test will be stratified by HIV status and TBM disease severity at presentation. Patients lost to follow up will be analyzed according to their last recorded disability status. If the rate of patients lost to follow-up exceeds 10%, we will also perform an alternative analysis based on multiple imputation of missing values.

Time-to-event endpoints, i.e. time to new neurological event or death and time to new or recurrent AIDS defining illness or death (in HIV positive patients only), will be analyzed with a log-rank test, Kaplan-Meier curves and Cox regression models as described for the primary endpoint above.

All reported serious and grade 3&4 adverse reactions will be listed; their overall frequencies and the rate of treatment interruptions due to adverse events will be compared between the two treatment groups using a generalized Cochran-Mantel-Haenszel test stratified by HIV status and TBM disease severity at presentation.

### Analysis populations

All patients will be analyzed in the primary analysis according to their randomization arm (intention-to-treat, ITT). The primary endpoint, overall survival, will in addition be analyzed on the per-protocol (PP) population which excluded the following patients: patients with a final diagnosis other than TBM, major protocol violations and those receiving less than 2 months of administration of the randomized study drug for reasons other than death.

## Ethical issues

### Ethical approval

This protocol, the patient information sheet, the patient consent form has been reviewed and approved by the Oxford Tropical Research Ethics Committee (OxTREC) and the Institutional Review Boards of the Hospital for Tropical Diseases and Pham Ngoc Thach Hospital. The study and study materials have also been submitted for approval by the Ethical Committee of the Ministry of Health Vietnam.

### Informed consent and information sheet

A patient cannot enter the trial without informed consent.

Written informed consent will be sought for all patients entering the trial. When written consent is not possible verbal consent will be considered acceptable in the presence of a witness who can attest to the accurate reading of the informed consent form and the agreement of the patient. The doctor entering the patient into the trial is responsible for obtaining informed consent. If the patient is unconscious, the consent of the relatives or family members is acceptable. If there are no relatives, the consent of two independent physicians will be considered acceptable. In this case consent from the patient will be sought as soon as the patient regains the ability to give or refuse consent.

### Withdrawal from the trial

Patients may voluntarily withdraw from the trial for any reason. If this occurs, the trial researchers are under no obligation to provide treatment. The withdrawal of the patient from the trial will not affect their access to the best standard of care within the national health system. Clinical and laboratory assessment should be performed and recorded at the time of withdrawal.

### Confidentiality

A unique trial number will be assigned to each patient entering the trial and will be used to identify all laboratory specimens and the case record forms. All records will be stored securely on the wards or in the OUCRU. Clinical information will not be released without written permission of the patient.

### Interim analysis and role of the Data and Safety Monitoring Committee (DSMC)

An independent DSMC will oversee the trial. Interim analyses are planned after 20 deaths have been observed, after 6 and 12 months of recruitment and yearly thereafter until the completion of the trial. The DSMC will be provided with unblinded summary tables of grade 3&4 and serious adverse events and an analysis of overall survival. These analyses will be performed by an independent statistician not otherwise involved with the trial.

Based on these data, the committee will make one of the following recommendations:

• Continue the trial without modification

• Continue the trial with modification

• Stop the trial due to safety concerns

Unless the benefit of intensified treatment is shown "beyond reasonable doubt" at an interim analysis, no formal stopping for efficacy is foreseen. The Haybittle-Peto boundary, requiring p < 0.001 at interim analysis to consider stopping for efficacy, should be used as a guidance. However, the DSMB recommendation should not be based purely on statistical tables but also requires clinical judgment. As the dissemination of preliminary summary data could influence the further conduct of the trial and introduce bias, access to interim data and results will be confidential and strictly limited to the involved independent statistician and the monitoring board and results (except for the recommendation) will not be communicated to the outside and/or clinical investigators involved in the trial.

## Discussion

Currently very few options are available for the treatment of TBM. There are 5 "first-line drugs" and a small number of "second-line drugs". With the exception of fluoroquinolones, the second-line drugs are relatively toxic and apart from ethionamide, cycloserine and some of the fluoroquinolones, penetration into the CSF is poor. Several new agents are now in the early stages of clinical evaluation, but will not be evaluated in treating TBM in the immediate future. The amplification of MDR-TB strains globally and the exceptionally high mortality among MDR-TBM patients are worrying signs of insufficient TB and TBM treatment globally. This trial is based on the hypothesis that current anti-mycobacterial treatment schedules for TBM are not potent enough and that outcomes will be improved by increasing the CSF penetrating power of this regimen by optimising dosage and using additional drugs with better CSF penetration. We acknowledge the fact that this trial is testing this hypothesis by essentially including two interventions in one arm of the trial. From a clinical point of view the main interest of this research is improving treatment, however scientifically it would be satisfying to know, if positive results are observed, to which drug they can be attributed or which modifications are strictly necessary in this treatment regimen.

In order to assess the effect of either intervention alone and the combined effect it is necessary to either perform a series of trials or do a 2 × 2 factorial trial. In a companion paper to the present study protocol [41] we show that an adequately powered 2 × 2 factorial design would require an eight-fold increase in sample size that would transform our study protocol from what will be the largest trial ever conducted in TBM to an impossible study. Currently 40% of all adult patients with TBM die from the disease. In view of this high mortality we argue for a pragmatic approach. The quest for optimal treatment should no longer be postponed. Subsequent trials to further refine the optimal treatment can be initiated if the present working hypothesis proves successful.

## Abbreviations

Ab: Antibody; AIDS: Acquired immune deficiency syndrome; ALT: Alanine aminotransferase; ARV: Antiretroviral; AST: Aspartate transaminase; AUC: Area Under the Curve; BTS: British Thoracic Society. CRF: Case record form; CSF: Cerebrospinal fluid; CT: Computed tomography; DIH: Drug Induced hepatitis; DNA: Deoxyribonucleic acid; DSMC: Data and safety monitoring committee; E: Ethambutol; EBA: Early bactericidal Activity; H: Isoniazid; HbsAg: Hepatitis B surface antigen; HBV: Hepatitis B virus; HCMC: Ho Chi Minh City; HCV: Hepatitis C virus; HIV: Human Immunodeficiency virus; HTD: Hospital for Tropical Diseases; ICH: International Conference on Harmonization; MDR TB: Multi-drug resistant tuberculosis; MIC: Minimum inhibitory concentration; MRC: Medical Research Council; MRI: Magnetic resonance imaging; Mtb: Mycobacterium tuberculosis; NICE: National Institute for Health and Clinical Excellence UK; NNRTI: Non- Nucleoase reverse transcriptase inhibitor; NRTI: Nucleoase reverse transcriptase inhibitor; NTP: National Tuberculosis Programme of Vietnam; NVP: Nevirapine; OPC: Out Patient clinic; OPD: Out Patient department; OUCRU: Oxford University Clinical Research Unit; OXTREC: Oxford Tropical Research Ethics Committte; PNT: Pham Ngoc Thach Hospital for Tuberculosis and Lung Diseases; R: Rifampicin; PEPFAR: President's Emergency Plan for AIDS Relief; RNA: Ribonucleic acid; S: Streptomycin; SAE: Severe adverse event; TB: Tuberculosis; TBM: Tuberculous meningitis; UK: United Kingdom; ULN: Upper limit of normal; US: United States; Z: Pryrazinamide; ZN: Ziehl-Neelsen.

## Competing interests

The authors declare that they have no competing interests.

## Authors' contributions

JF, DH, JD, TTHC, NTBY, MC, MW conceived the study. All authors discussed the development of the protocol and the manuscript and approved the final version.

## Supplementary Material

Additional file 1 **Diagnostic criteria for tuberculous meningitis**. Diagnosis and grading of tuberculous meningitis, including outcome and disability. Click here for file

Additional file 2 **Outcome and disability grading**. Click here for file

Additional file 3 **Toxicity grading and management**. Table of common toxicity criteria. Click here for file

Additional file 4 **Modified MRC grading for tuberculous meningitis**. Click here for file

Additional file 5 **Standard TBM treatment**. First-line antituberculous therapy. Click here for file

Additional file 6 **Dexamethasone therapy**. Click here for file

Additional file 7 **Guide to management of toxicities**. Click here for file

Additional file 8 **Management of common adverse effects of antituberculous medications**. Click here for file

Additional file 9 **Reintroduction of antituberculous therapy**. Based on British Thoracic Society Guidelines for chemotherapy and management of tuberculosis (Thorax 1998; 53: 536-548). Click here for file
