# Supplementary material for: Intensified treatment with high dose Rifampicin and Levofloxacin compared to standard treatment for adult patients with Tuberculous Meningitis (TBM-IT): protocol for a randomized controlled trial
Source: Trials. 2011 Feb 2;12:25. doi: 10.1186/1745-6215-12-25 (PMC3041687; doi:10.1186/1745-6215-12-25)
Supplement: Additional file 1 — Diagnostic criteria for tuberculous meningitis. Diagnosis and grading of tuberculous meningitis, including outcome and disability. [file 1745-6215-12-25-S1.DOC]

Diagnosis and grading of tuberculous meningitis, including outcome and disability

Diagnostic criteria for tuberculous meningitis

| Classification | Diagnostic criteria |
| --- | --- |
| Definite TBM | Clinical meningitis plus acid-fast bacilli seen in the CSF or  *M. tuberculosis* cultured from the CSF |
| Probable TBM | Clinical meningitis plus one of the following criteria:   - Radiographic evidence of pulmonary tuberculosis - Acid-fast bacilli seen in sputum or gastric fluid - Evidence of extra-pulmonary tuberculosis - CT or MRI brain scan features consistent with TBM |
| Possible TBM | Clinical meningitis plus ≥ 2 of the following criteria:   - History of previous tuberculosis - Illness duration > 5 days - Glasgow coma score < 15 - Focal neurological signs   and ≥ 2 of the following criteria:   - Yellow CSF - > 50% lymphocytes in the CSF - CSF glucose < 50% blood glucose |
