# Supplementary material for: Intensified treatment with high dose Rifampicin and Levofloxacin compared to standard treatment for adult patients with Tuberculous Meningitis (TBM-IT): protocol for a randomized controlled trial
Source: Trials. 2011 Feb 2;12:25. doi: 10.1186/1745-6215-12-25 (PMC3041687; doi:10.1186/1745-6215-12-25)
Supplement: Additional file 2 — Outcome and disability grading. [file 1745-6215-12-25-S2.DOC]

Outcome and disability grading

The ‘Two simple questions’

| Does the patient require help from anybody for everyday activities? *(For example eating, drinking, washing,brushing teeth, going to the toilet.)* | Yes/No | Yes= Poor outcome |
| --- | --- | --- |
| Has the illness left you with any other problems? | Yes/No | Yes= Indifferent outcome  No= Good outcome |

The Modified Rankin Scale

| grade | Description |
| --- | --- |
| 0 | No symptoms |
| 1 | Minor symptoms not interfering with lifestyle |
| 2 | Symptoms that lead to some restriction in lifestyle, but do not interfere with the patients ability to look after themselves |
| 3 | Symptoms that restrict lifestyle and prevent totally independent living |
| 4 | Symptoms that clearly prevent independent living, although the patient does not need constant care and attention. |
| 5 | Totally dependent, requiring constant help day and night. |
