# Supplementary material for: Intensified treatment with high dose Rifampicin and Levofloxacin compared to standard treatment for adult patients with Tuberculous Meningitis (TBM-IT): protocol for a randomized controlled trial
Source: Trials. 2011 Feb 2;12:25. doi: 10.1186/1745-6215-12-25 (PMC3041687; doi:10.1186/1745-6215-12-25)
Supplement: Additional file 3 — Toxicity grading and management. Table of common toxicity criteria. [file 1745-6215-12-25-S3.DOC]

Toxicity grading and management

Table of common toxicity criteria

Note: ULN = upper limit of normal local reference range

|  | Grade 1 | Grade 2 | Grade 3 | Grade 4 |
| --- | --- | --- | --- | --- |
| Haematological |  |  |  |  |
| Haemoglobin | 8.0 - 9.4 g/dl | 7.0 - 7.9 g/dl | 6.5 –6.9g/dl | <6.5 g/dl |
| White cell count | 3.0 - 3.9 x 10^3^ cells/μl | 2.0 - 2.9 x 10^3^ cells/μl | 1.0 - 1.9 x 10^3^ cells/μl | <1.0 x 10^3^ cells/μl |
| Neutrophils | 1.0 – 1.5 x 10^3^ cells/μl | 0.75 – 0.99 x 10^3^ cells/μl | 0.5 – 0.74 x 10^3^ cells/μl | <0.5 x 10^3^ cells/μl |
| Platelets | 75 - 99 x 10^3^ cells/μl | 50 - 74 x 10^3^ cells/μl | 20 - 49 x 10^3^ cells/μl | <20 x 10^3^ cells/μl |
| Prothrombin time | >1.0 – 1.25 x ULN | >1.25 – 1.5 x ULN | >1.5 – 3.0 x ULN | >3.0 x ULN |
| Biochemical |  |  |  |  |
| Hyponatraemia | 130 – 135 mmol/l | 123-129 mmol/l | 116-122 mmol/l | <116 mmol/l |
| Hypernatraemia | 146 – 150 mmol/l | 151 – 157 mmol/l | 158 – 165 mmol/l | >165 mmol/l |
| Hypokalaemia | 3.0 – 3.4 mmol/l | 2.5 – 2.9 mmol/l | 2.0 – 2.4 mmol/l | <2.0 mmol/l |
| Hyperkalaemia | 5.6 – 6.0 mmol/l | 6.1 – 6.5 mmol/l | 6.6 – 7.0 mmol/l | >7.0 mmol/l |
| Hypoglycaemia | 3.1 – 3.6 mmol/l  55 – 64 mg/dl | 2.2 – 3.0 mmol/l  40-54 mg/dl | 1.7 – 2.1 mmol/l  30 – 39 mg/dl | <1.7 mmol/l  <30 mg/dl |
| Hyperglycaemia (fasting) | 6.5 – 9.0 mmol/l  118 – 164 mg/dl | 9.1 – 14.0 mmol/l  165 – 255 mg/dl | 14.1 – 28.0 mmol/l  256 – 509 mg/dl | >28.0 mmol/l, >509 mg/dl or ketoacidosis |
| Urea | 1.25 – 2.5 x ULN | >2.5 – 5.0 x ULN | >5.0 – 10.0 x ULN | >10.0 x ULN |
| Creatinine | >1.0 – 1.5 x ULN | >1.5 – 3.0 x ULN | >3.0 – 6.0 x ULN | >6.0 x ULN |
| Bilirubin | >1.0 – 1.5 x ULN | >1.5 – 2.5 x ULN | >2.5 – 5.0 x ULN | >5.0 x ULN |
| AST or ALT or GGT | 1.25 – 2.5 x ULN | >2.5 – 5.0 x ULN | >5.0 – 10.0 x ULN | >10.0 x ULN |
| Alkaline phosphatase | 1.25 – 2.5 x ULN | >2.5 – 5.0 x ULN | >5.0 – 10.0 x ULN | >10.0 x ULN |
| Amylase | >1.0 – 1.5 x ULN | >1.5 – 2.5 x ULN | >2.5 – 5.0 x ULN | >5.0 x ULN |
| Urinalysis |  |  |  |  |
| Haematuria | Microscopic | Gross, no clots | Gross and clots | Obstruction or requiring transfusion |
| Proteinuria | 1+ or <0.3% or <3g/l or 200mg-1g loss/day | 2-3+ or 0.3 -1.0% or 3-10g/l or 1-2g loss/day | 4+ or >1.0% or >10g/l or 2-3.5g loss/day | Nephrotic syndrome or  >3.5g loss/day |

Table of common toxicity criteria (cont’d)

|  | Grade 1 | Grade 2 | Grade 3 | Grade 4 |
| --- | --- | --- | --- | --- |
| Stomatitis/mouth ulcers | Mild discomfort, no limits on activity | Some limits on eating or talking | Eating/talking very limited | Requiring IV fluids |
| Nausea | Mild or transient discomfort, maintains reasonable intake | Moderate discomfort or significantly decreased intake for > 3 days | Severe discomfort or minimal intake for ≥ 3 days | Hospitalization required |
| Vomiting | Mild or transient, 2-3 episodes per day or mild vomiting lasting < 1 week | Moderate or persistent, 4-5 episodes/day or vomiting lasting ≥ 1 week | Severe vomiting of all foods/fluids in 24 hours or orthostatic hypotension or IV fluids required | Hypotensive shock or hospitalization required for IV fluids |
| Diarrhoea | Mild or transient, 3-4 loose stools/day or mild diarrhoea lasting < 1 week | Moderate or persistent, 5-7 loose stools per day or diarrhoea lasting ≥ 1 week or nocturnal loose stools | Bloody diarrhoea or orthostatic hypotension or ≥ 7 loose stools per day or requiring IV fluids | Hypotensive shock or hospitalization required for IV fluids |
| Clinical pancreatitis | Mild abdominal pain, amylase < 2.5 x ULN, other causes excluded | Moderate abdominal pain, amylase <2.5x ULN, other causes excluded | Severe abdominal pain, amylase > 2.5 x ULN, hospitalization required. | Severe abdominal pain, shock/hypovolaemia, amylase > 5 x ULN, hospitalization required |
| Headache | Mild, no treatment | Moderate or requires non-narcotic analgesia | Severe or responds to first narcotic | Intractable or requiring repeated narcotics |
| Consciousness | Difficulty in concentration or memory | Mild confusion or lethargy <50% waking hours | Disorientation or stupor >50% of waking hours | Coma or seizures |
| Mood | Mild anxiety or depression | Treatment required for anxiety or depression | Treatment and assistance required, severe depression, mania or anxiety | Acute psychosis or hospitalization |
| Psychosis | Mild agitation or confusion | Some limitation in activities of daily living and minimal treatment required | Treatment and assistance required, severe agitation or confusion | Toxic psychosis or hospitalization |
| Cerebellar | Slight incoordination or dysdiadichokinesia | Intention tremor or dysmetria or slurred speech or nystagmus | Ataxia requiring assistance to walk or arm incoordination interfering with activities of daily living | Unable to stand |

Table of common toxicity criteria (cont’d)

|  | Grade 1 | Grade 2 | Grade 3 | Grade 4 |
| --- | --- | --- | --- | --- |
| Neurological |  |  |  |  |
| Motor | Mild weakness in feet but able to walk or mild increase or decrease in reflexes | Moderate weakness in feet (unable to walk on heels or toes), mild weakness in hands but still able to do most hand tasks, or loss of previously present reflex or development  of hyperreflexia or unable to do deep knee bends due to  weakness | Marked distal weakness (unable to dorsiflex toes or foot drop) and moderate  proximal weakness (e.g. in hands interfering with activities of daily living or requiring assistance to walk or unable to rise from chair unassisted) | Confined to bed or wheelchair because of muscle weakness |
| Clinical myopathy | Minimal findings | Moderate myalgia or difficulty climbing stairs or rising from sitting position, able to walk, may need NSAID | Moderate to severe myalgia needing NSAID, assistance  required for walking or  general activities | Severe myalgia unrelated to exercise requiring narcotics,  unable to walk or necrosis or oedema |
| Sensory | Mild impairment (decreased sensation e.g. vibratory, pinprick, hot/cold in great toes) in focal area or  symmetrical distribution | Moderate impairment (moderately decreased  sensation e.g. vibratory, pinprick, hot/cold to ankles) or joint position or mild impairment that is not symmetrical | Severe impairment (decrease or loss of sensation to knees or wrists) or loss of sensation of moderate degree in multiple different body areas (e.g. upper and lower  extremities) | Sensory loss involves limbs and trunk |
| Parasthaesia | Mild discomfort, no treatment | Moderate discomfort,  requiring non-narcotic analgesia | Severe discomfort or symptoms respond to narcotic analgesia | Incapacitating or not responsive to narcotics |
| Peripheral neuropathy | Mild paraesthesia, numbness, pain or weakness, not treated | Moderate paraesthesia,  numbness or pain, objective weakness, requires analgesic | Severe, narcotic required, interferes with normal activity | Intolerable, incapacitating, unable to walk despite narcotics, paralysis |
| Respiratory |  |  |  |  |
| Bronchospasm | Transient, no treatment,  70-80% peak flow or FEV1 | Requires treatment, normalizes with bronchodilator, 50-69% peak flow or FEV1 | No normalization with bronchodilator, 25-49% peak flow or FEV1, retractions | Cyanosis, intubated or <25% peak flow or FEV1 |

Table of common toxicity criteria (cont’d)

|  | Grade 1 | Grade 2 | Grade 3 | Grade 4 |
| --- | --- | --- | --- | --- |
| Cardiovascular |  |  |  |  |
| Cardiac arrhythmia |  | Asymptomatic, transient dysrhythmia, no treatment | Recurrent or persistent  dysrhythmia, symptomatic, treatment required | Unstable dysrhythmia,  hospitalization and treatment required |
| Hypertension | Transient, increase >20mm/Hg, no treatment | Recurrent, chronic increase >20mm/Hg, requires treatment | Acute treatment required, outpatient, hospitalization possible | Hospitalization required |
| Hypotension | Transient, orthostatic hypotension, no treatment | Symptoms correctable with oral fluid treatment | IV fluid required, no hospitalization required | Hospitalization required |
| Pericarditis | Minimal effusion | Mild/moderate asymptomatic effusion, no treatment | Symptomatic effusion, pain, ECG changes | Tamponade or pericardiocentesis or surgery required |
| Haemorrhage | Microscopic or occult | Mild, no transfusion | Gross blood loss or transfused 1-2 units | Massive blood loss or transfused >2 units |
| Other |  |  |  |  |
| Fever, oral, > 12 hours | 37.7-38.5°C | 38.6-39.5°C | 39.6-40.5°C | >40.5°C |
| Fatigue | Normal activity reduced by <25% | 25-50% decrease in normal activity | >50% decrease in activity, | Cannot work, unable to care for self |
| Hypersensitivity | Pruritus without rash | Localized urticaria | Generalised urticaria or  angioedema | Anaphylaxis |
| Rash | Rash, erythema or pruritus | Diffuse maculopapular rash or dry desquamation | Vesiculation or moist desquamation or ulceration | Exfoliative dermatitis or mucous membrane involve-ment or suspected Stevens-Johnson or erythema  multiforme or necrosis requiring surgery |
| General | General mild, transient, easily  tolerated, no treatment | Moderate, discomfort, interrupts usual activity, may require minor treatment | Severe, considerable interference with usual activity, requires treatment or intervention | Incapacitating or lifethreatening,  requires treatment and/or  hospitalization |
