# Supplementary material for: Intensified treatment with high dose Rifampicin and Levofloxacin compared to standard treatment for adult patients with Tuberculous Meningitis (TBM-IT): protocol for a randomized controlled trial
Source: Trials. 2011 Feb 2;12:25. doi: 10.1186/1745-6215-12-25 (PMC3041687; doi:10.1186/1745-6215-12-25)
Supplement: Additional file 4 — Modified MRC grading for tuberculous meningitis. [file 1745-6215-12-25-S4.DOC]

Modified MRC grading for tuberculous meningitis

| TBM grade | Diagnostic criteria |
| --- | --- |
| Grade I | Glasgow coma score 15, no focal neurology |
| Grade II | Glasgow coma score 11-14 OR  Glasgow coma score 15 with focal neurology |
| Grade III | Glasgow coma score ≤ 10 |
