# Supplementary material for: Intensified treatment with high dose Rifampicin and Levofloxacin compared to standard treatment for adult patients with Tuberculous Meningitis (TBM-IT): protocol for a randomized controlled trial
Source: Trials. 2011 Feb 2;12:25. doi: 10.1186/1745-6215-12-25 (PMC3041687; doi:10.1186/1745-6215-12-25)
Supplement: Additional file 5 — Standard TBM treatment. First-line antituberculous therapy. [file 1745-6215-12-25-S5.DOC]

**Standard TBM treatment**

**First-line antituberculous therapy**

| **Drug** | **Dose** |
| --- | --- |
| Iso niazid (H) | 5mg/kg od po, max 300mg/day |
| Ri fampicin (R) | 10mg/kg od po max dose 750mg/day |
| Py razinamide (Z) | 25mg/kg od po, max 2g/day |
| Et hambutol (E) and/or | 20mg/kg od po, max 1.2g/day |
| Str eptomycin (S) | 20mg/kg od im, max 1g/day |

After 3 months, pyrazinamide and ethambutol/streptomycin will be stopped and the patient will continue on rifampicin and isoniazid at the same doses for a further six months.
