# Supplementary material for: Intensified treatment with high dose Rifampicin and Levofloxacin compared to standard treatment for adult patients with Tuberculous Meningitis (TBM-IT): protocol for a randomized controlled trial
Source: Trials. 2011 Feb 2;12:25. doi: 10.1186/1745-6215-12-25 (PMC3041687; doi:10.1186/1745-6215-12-25)
Supplement: Additional file 6 — Dexamethasone therapy. [file 1745-6215-12-25-S6.DOC]

**Dexamethasone therapy**

|  | Grade I TBM | **Grades II and III TBM** |
| --- | --- | --- |
| Week 1 | 0.3 mg/kg iv | 0.4 mg/kg iv |
| Week 2 | 0.2 mg/kg iv | 0.3 mg/kg iv |
| Week 3 | 0.1 mg/kg iv | 0.2 mg/kg iv |
| Week 4 | 3.0mg total/day po | 0.1 mg/kg iv |
| Week 5 | 2.0mg total/day po | 4.0mg total/day po |
| Week 6 | 1.0mg total/day po | 3.0mg total/day po |
| Week 7 |  | 2.0 mg total/day po |
| Week 8 |  | 1.0 mg total/day po |
