# Supplementary material for: Intensified treatment with high dose Rifampicin and Levofloxacin compared to standard treatment for adult patients with Tuberculous Meningitis (TBM-IT): protocol for a randomized controlled trial
Source: Trials. 2011 Feb 2;12:25. doi: 10.1186/1745-6215-12-25 (PMC3041687; doi:10.1186/1745-6215-12-25)
Supplement: Additional file 7 — Guide to management of toxicities. [file 1745-6215-12-25-S7.DOC]

Guide to management of toxicities

Grade 1 clinical or laboratory toxicities

- Continue study drugs

Grade 2 clinical or laboratory toxicities

- Continue study drugs
- If relevant, monitor more closely and consider more frequent laboratory assessments
- Investigate to exclude other causes

Grade 3 clinical or laboratory toxicities

- Monitor more closely
- Perform more frequent laboratory assessments
- Investigate to exclude other causes
- For AST or ALT > 5 x ULN stop all study drugs until toxicity resolves and consider reintroduction of antituberculous drugs sequentially.
- For other grade 3 toxicities the clinician may immediately stop study drugs if confirmatory test cannot be performed within 72 hours or if the clinician determines that continuation of study drugs is unsafe while awaiting test results
- Fill in an adverse event form and inform the DSMC

Grade 4 clinical or laboratory toxicities

- Monitor more closely
- Perform more frequent laboratory assessments
- Investigate to exclude other causes
- For all grade 4 toxicities that are attributable to antituberculous drugs, stop all drugs until toxicity resolves and restart antituberculous drugs sequentially
- For all grade 4 toxicities that are clearly attributable to antiretroviral drugs, stop relevant drugs until toxicity resolves and consider switching to alternative drugs
- For other grade 4 toxicities the clinician may immediately stop study drugs if confirmatory test cannot be performed within 72 hours or if the clinician determines that continuation of study drugs is unsafe while awaiting test results
- If any doubt about management discuss with the principal investigator
- Fill in an adverse event form and inform the DSMC
