# Supplementary material for: Intensified treatment with high dose Rifampicin and Levofloxacin compared to standard treatment for adult patients with Tuberculous Meningitis (TBM-IT): protocol for a randomized controlled trial
Source: Trials. 2011 Feb 2;12:25. doi: 10.1186/1745-6215-12-25 (PMC3041687; doi:10.1186/1745-6215-12-25)
Supplement: Additional file 8 — Management of common adverse effects of antituberculous medications. [file 1745-6215-12-25-S8.DOC]

Management of common adverse effects of antituberculous medications

| Adverse effect | Management |
| --- | --- |
| Gastrointestinal symptoms | Common in the first few weeks of treatment. Liver function tests should be checked and if the AST < 2 x ULN, the symptoms are assumed not to be due to hepatic toxicity. The initial management is to change the hour of drug administration and/or to administer the drugs with food. |
| Rash | If mild, affecting only a limited area or predominantly causing itching an antihistamine may be given for symptomatic relief and antituberculous medications may be continued. A petechial rash may be caused by rifampicin induced thrombocytopaenia – check platelet count and, and if low, stop rifampicin permanently. If there is a generalized erythematous rash, especially if associated with fever and/or mucous membrane involvement, stop all drugs. Once the rash has improved restart antituberculous drugs according to Table 7.5 |
| Drug fever | Fever may persist for 2 months after treatment has been initiated. Recurrence of fever in a patient who has been on therapy for several weeks may be due to drug fever, especially if the patient is showing clinical and microbiological improvement. Fever may also be a feature of immune reconstitution syndrome or other HIV-related infections. Potential causes should be excluded before stopping antituberculous drugs – drug fever usually resolves in 24 hours. Once the fever has resolved restart drugs according to Table 7.5 |
| Hepatitis | Isoniazid, rifampicin or pyrazinamide can all cause drug-induced liver injury Asymptomatic increases in AST occurs in around 20% of patients treated with 4 drugs and most resolve spontaneously. The frequency of clinical and laboratory monitoring should increase but therapy should not be altered. However, if AST or ALT >5 x ULN all hepatotoxic drugs should be stopped. The patient should be evaluated for other causes (viral hepatitis, alcohol intake, other hepatotoxins, biliary tract disease) before diagnosing drug-induced hepatitis. Once symptoms have resolved and AST returns to < 2 x ULN antituberculous medications may be restarted |
