# Supplementary material for: Intensified treatment with high dose Rifampicin and Levofloxacin compared to standard treatment for adult patients with Tuberculous Meningitis (TBM-IT): protocol for a randomized controlled trial
Source: Trials. 2011 Feb 2;12:25. doi: 10.1186/1745-6215-12-25 (PMC3041687; doi:10.1186/1745-6215-12-25)
Supplement: Additional file 9 — Reintroduction of antituberculous therapy. Based on British Thoracic Society Guidelines for chemotherapy and management of tuberculosis (Thorax 1998; 53: 536-548). [file 1745-6215-12-25-S9.DOC]

Reintroduction of antituberculous therapy

Based on British Thoracic Society Guidelines for chemotherapy and management of tuberculosis (Thorax 1998; 53: 536-548)

| **Drug** | **Day 0** | **Day 3** | **Day 6** | **Day 9** | **Day 12** | **Day 15** | **Day 18** | **Day 21** | **Day 24** |
| --- | --- | --- | --- | --- | --- | --- | --- | --- | --- |
| Isoniazid 50mg | X |  |  |  |  |  |  |  |  |
| Isoniazid 300mg |  | X | X | X | X | X | X | X | X |
| Rifampicin 75mg |  |  | X |  |  |  |  |  |  |
| Rifampicin 300mg |  |  |  | X |  |  |  |  |  |
| Rifampicin 450mg |  |  |  |  | X |  |  |  |  |
| Rifampicin 600mg |  |  |  |  |  | X | X | X | X |
| Pyrazinamide 250mg |  |  |  |  |  |  | X |  |  |
| Pyrazinamide 1g |  |  |  |  |  |  |  | X |  |
| Pyrazinamide 1.5g  (if wt < 50kg) |  |  |  |  |  |  |  |  | X |
| Pyrazinamide 2g  (if wt ≥ 50kg) |  |  |  |  |  |  |  |  | X |

Notes

1. Closely monitor patient’s clinical condition and liver function tests while reintroducing drugs
2. If no reaction occurs to a new drug then increase the dose to maximum or add the next drug every 3 days, according to the table
3. If a reaction occurs, stop the offending drug and await resolution of symptoms
4. If pyrazinamide found to be the offending drug, extend treatment period and continue ethambutol for initial 3 months
